# Interleukin-22 is increased in multiple sclerosis patients and targets astrocytes

**DOI:** 10.1186/s12974-015-0335-3

**Published:** 2015-06-16

**Authors:** Guillaume Perriard, Amandine Mathias, Lukas Enz, Mathieu Canales, Myriam Schluep, Melanie Gentner, Nicole Schaeren-Wiemers, Renaud A. Du Pasquier

**Affiliations:** Laboratory of Neuroimmunology, Center of Research in Neurosciences, Department of Clinical Neurosciences and Service of Immunology and Allergy, Department of Medicine, CHUV, 1011 Lausanne, Switzerland; Service of Neurology, Department of Clinical Neurosciences, CHUV BH-10/131, 46, rue du Bugnon, 1011 Lausanne, Switzerland; Neurobiology, Department of Biomedicine, University Hospital Basel, University of Basel, 4031 Basel, Switzerland

**Keywords:** Multiple sclerosis, Interleukin-22, Astrocytes, Survival

## Abstract

**Background:**

Increasing evidences link T helper 17 (Th17) cells with multiple sclerosis (MS). In this context, interleukin-22 (IL-22), a Th17-linked cytokine, has been implicated in blood brain barrier breakdown and lymphocyte infiltration. Furthermore, polymorphism between MS patients and controls has been recently described in the gene coding for IL-22 binding protein (IL-22BP). Here, we aimed to better characterize IL-22 in the context of MS.

**Methods:**

IL-22 and IL-22BP expressions were assessed by ELISA and qPCR in the following compartments of MS patients and control subjects: (1) the serum, (2) the cerebrospinal fluid, and (3) immune cells of peripheral blood. Identification of the IL-22 receptor subunit, IL-22R1, was performed by immunohistochemistry and immunofluorescence in human brain tissues and human primary astrocytes. The role of IL-22 on human primary astrocytes was evaluated using 7-AAD and annexin V, markers of cell viability and apoptosis, respectively.

**Results:**

In a cohort of 141 MS patients and healthy control (HC) subjects, we found that serum levels of IL-22 were significantly higher in relapsing MS patients than in HC but also remitting and progressive MS patients. Monocytes and monocyte-derived dendritic cells contained an enhanced expression of mRNA coding for IL-22BP as compared to HC.

Using immunohistochemistry and confocal microscopy, we found that IL-22 and its receptor were detected on astrocytes of brain tissues from both control subjects and MS patients, although in the latter, the expression was higher around blood vessels and in MS plaques.

Cytometry-based functional assays revealed that addition of IL-22 improved the survival of human primary astrocytes. Furthermore, tumor necrosis factor α-treated astrocytes had a better long-term survival capacity upon IL-22 co-treatment. This protective effect of IL-22 seemed to be conferred, at least partially, by a decreased apoptosis.

**Conclusions:**

We show that (1) there is a dysregulation in the expression of IL-22 and its antagonist, IL-22BP, in MS patients, (2) IL-22 targets specifically astrocytes in the human brain, and (3) this cytokine confers an increased survival of the latter cells.

**Electronic supplementary material:**

The online version of this article (doi:10.1186/s12974-015-0335-3) contains supplementary material, which is available to authorized users.

## Background

Multiple sclerosis (MS) occurs in genetically predisposed young adults with probable environmental triggers [1]. Infiltrating auto-reactive immune cells, in synergy with resident glial cells, will cause neuroinflammation and neurodegeneration, as characterized by demyelination, axonal loss, and finally irreversible damage to the central nervous system (CNS) [2]. Pro-inflammatory T helper 17 (Th17) cells have been associated with MS [3–9], but the function of Th17 cells in the pathogenesis of MS is still a matter of debate [10–12].

Interleukin-22 (IL-22), a Th17-linked cytokine, is associated with autoimmune diseases such as inflammatory bowel diseases and psoriasis [13]. Depending on the targeted tissue and the cytokine milieu in which it is released, IL-22 can contribute to inflammation, chemotaxis, and host defense but also to cell survival, tissue protection, wound healing, and epithelial cell proliferation [14–20]. IL-22 modulates immunity at barrier surface in multiple human diseases [21]. Together with IL-17, IL-22 seems to compromise the blood brain barrier integrity, enabling lymphocyte ingress into the CNS, which raises the possibility that this cytokine may contribute to MS severity [22]. The melanoma cell adhesion molecule (MCAM) has been associated with infiltration of T cells into CNS lesions together with an increased expression of IL-17 and IL-22 in MCAM^+^ T cells as compared to MCAM^−^ T cells [23, 24]. Somewhat contrasting with the previous findings, some data suggest that IL-22 may not necessarily be pro-inflammatory: in Theiler’s virus-induced demyelination in mice, epitope-specific CD8^+^ T cells causing minimal cytotoxicity in the CNS expressed a higher level of IL-22 mRNA than highly cytotoxic CD8^+^ T cells [25]. IL-22 may even be useful for tissue-protective therapy [26]. Further suggesting that IL-22 may be involved in MS, genetic studies showed that the gene coding for IL-22 binding protein (IL-22BP, also called IL-22RA2), an antagonist of IL-22 [27–30], harbored different single nucleotide polymorphism in MS patients as compared to control subjects [31–34]. Interestingly, this secreted IL-22 inhibitory receptor exacerbates experimental autoimmune encephalomyelitis (EAE) disease course [31, 35], raising the question whether IL-22 itself may have an anti-inflammatory function in EAE.

These data suggest that IL-22 may be involved in the immunopathogenesis of MS. However, this cytokine has been barely studied in MS patients. Here, we investigated whether IL-22 and IL-22BP are dysregulated in MS. We further aimed at identifying its target cells in human brain tissues, in particular in MS patients, and determining its functional effect in the CNS.

## Methods

### Study subjects

For the studies pertaining to the determination of IL-22 and IL-22BP levels in the blood, 141 MS patients and healthy control (HC) subjects were enrolled and divided into subgroups according to the disease type (Table 1). The diagnosis of MS followed the revised McDonald criteria [36]. The category of clinically active multiple sclerosis patients comprised relapsing remitting (RR)-MS or clinically isolated syndrome (CIS), who had a relapse that started less than 2 months prior to our assays. The category of clinically inactive multiple sclerosis patients included RR-MS and CIS patients who were in remission, as defined by an interval of more than 3 months after the last relapse. The category of progressive MS patient group contained patients with secondary progressive (SP)-MS or primary progressive (PP)-MS. Clinically inactive, SP- and PP-MS patients were not under any treatment within the 3 months prior to the blood draw. Among the 26 clinically active MS patients, four were on interferon-β, one on natalizumab, and one on fingolimod treatments. None of the MS patients had received corticosteroids in the previous 3 months. All enrolled patients and healthy control subjects signed an informed consent form, according to our institution review board.

**Table 1:** Study subjects for the assessment of IL-22 and IL-22BP in the blood

Category	*N* = 141	Male/female ratio	Age at blood draw (years)^a^	EDSS^a^	Disease duration (years)^a^	Last relapse (months)^a^
Clinically active MS patients	26	7/19	31.5 ± 10.0	2.00 ± 1.00	0.63 ± 7.19	0.46 ± 0.82
Clinically inactive MS patients	35	10/25	39.5 ± 11.5	1.50 ± 0.63	7.00 ± 9.25	16.87 ± 34.43
Progressive MS patients	35	12/23	52.0 ± 15.0	4.50 ± 2.50	17.0 ± 12.0	–
Healthy control subjects	45	21/24	34.0 ± 28.5	–	–	–

*MS* multiple sclerosis, *EDSS* expanded disability status scale

^a^Median ± interquartile range

For immunohistochemistry studies, brain biopsies from 11 non-MS patients were obtained from neurosurgical resections performed in the service of neurosurgery at the CHUV in Lausanne, hereafter referred to as the “Lausanne cohort” (Table 2). Tissues from five MS patients with their seven control counterparts obtained after postmortem autopsies were processed in Basel and named “Basel cohort” (Table 2). All human brain tissues were collected in accordance with local ethical committee from the University Hospitals of Lausanne and Basel and the UK MS Tissue Bank.

**Table 2:** Study subjects for the assessment of IL-22 and IL-22 receptor in the brain

Patient	Gender	Age at surgery (years)	Type of surgery	Neuropathology reported from autopsy	Cause of surgery/death	Postmortem time (hours)	MS type	Disease duration (years)
**Lausanne cohort**								
L-C1	F	60	Biopsy	–	Cerebellar softening	–	–	–
L-C2	M	31	Biopsy	–	Epilepsy	–	–	–
L-C3	F	51	Biopsy	–	Hematoma	–	–	–
L-C4	M	43	Biopsy	–	Aneurysm	–	–	–
L-C5	M	41	Biopsy	–	Hematoma	–	–	–
L-C6	n/a	n/a	Biopsy	–	Polymorphic neuroectodermal tumor	–	–	–
L-C7	n/a	n/a	Biopsy	–	Glioblastoma	–	–	–
L-C8	M	32	Biopsy	–	Cavernoma	–	–	–
L-C9	F	53	Biopsy	–	Epilepsy	–	–	–
L-C10	M	51	Biopsy	–	Malformation	–	–	–
L-C11	M	39	Biopsy	–	Epilepsy	–	–	–
**Basel cohort**								
B-C1	M	75	Autopsy	Many documented neuropathological findings	Cerebrovascular accident, aspiration pneumonia	17	–	–
B-C2	M	64	Autopsy	Occasional hypoxic neurons, perineuronal oedema, stasis of erythrocytes in vessels, many leukocyte infiltrates	Cardiac failure	18	–	–
B-C3	M	84	Autopsy	Fibrosis of vessel walls, mild WM gliosis, perivascular oedema	Bladder cancer, pneumonia	5	–	–
B-C4	M	35	Autopsy	–	Carcinoma of the tongue	22	–	–
B-C5	M	64	Autopsy	Occasional hypoxic nerve cells, perineuronal oedema, fibrosis of the meninges	Cardiac failure	18	–	–
B-C6	F	84	Autopsy	Old cortical microinfarcts and acute global hypoxic changes. Senile changes are also present (amyloid deposits)	Congestive cardiac failure, ischemic heart disease, atrial fibrillation	24	–	–
B-C7	F	60	Autopsy	Brain with diffuse hypoxic changes	Ovarian cancer	13	–	–
B-MS1	M	40	Autopsy	No lesion, few leukocyte infiltrates	Respiratory failure, sepsis, MS	10	SP-MS	9
B-MS2	F	78	Autopsy	No lesion, some vessels with leukocyte infiltrates	Metastatic carcinoma of bronchus	5	SP-MS	42
B-MS3	F	34	Autopsy	Lesions in GM and WM, leukocytes around vessels	Pneumonia	12	SP-MS	n/a
B-MS4	F	49	Autopsy	Lesions in WM, leukocytes infiltrates	Bronchopneumonia, MS	7	SP-MS	21
B-MS5	M	44	Autopsy	n/a	Bronchopneumonia	16	SP-MS	n/a

*M* male, *F* female, *C* control, *MS* multiple sclerosis, *B* Basel, *L* Lausanne, *GM* gray matter, n/a not available, *SP* secondary progressive, *WM* white matter

### Human brain tissues

#### Lausanne cohort

In Lausanne, biopsied brain tissues were obtained only from non-MS study subjects. Just after biopsy, these ex vivo brain tissues, encompassing either white matter (WM), gray matter (GM), or both, were frozen and stored at −80 °C until they were cut to obtain 12-μm cryosections for immunofluorescence experiments. Hereafter, biopsied brain tissues from Lausanne cohort are named L-C1 to L-C11 (Lausanne-Control #1–11; Table 2).

#### Basel cohort

In Basel, brain tissues were obtained from postmortem autopsies supplied by the UK Multiple Sclerosis Tissue Bank at the Imperial College (UK Multicentre Research Ethics Committee, MREC/02/2/39), funded by the Multiple Sclerosis Society of Great Britain and Northern Ireland (registered charity 207,495). In addition to the brains of MS patients, there were also brain samples, including cortex and subcortical WM, from non-MS control patients (Table 2). Postmortem autopsy tissues were directly frozen and stored at −80 °C before use. Cryostat tissue sections (12 μm) from MS and control subject were mounted on Superfrost plus slides (Merck), dried for 30 min, and fixed with 4 % paraformaldehyde in phosphate-buffered saline (PBS) for 10 min at room temperature (RT). Slides were washed in PBS before staining. Brain tissues from Basel cohort are listed B-C1 to B-C7 and B-MS1 to B-MS5, referring to Basel control subjects and MS patients, respectively (Table 2).

### Primary human astrocytes

Human primary astrocytes (HA) derived from brain cerebral cortex were purchased from ScienCell Research Laboratory and were cultured according to the provider’s instructions. Briefly, HA were grown and cultured at 37 °C with 5 % CO_2_, in astrocyte medium (AM), supplemented with 2 % fetal calf serum (FCS) and 1 % astrocyte nutritive supplement with 1 % penicillin/streptomycin (referred as “complete astrocyte medium”). For immunofluorescence, cells were fixed for 15 min with 4 % paraformaldehyde and stored in phosphate-buffered saline (PBS) at 4 °C. For flow cytometry, cells were resuspended with trypsin (BioConcept) and first labeled with LIVE/DEAD fixable violet dead cell stain (Life Technologies). Then, HA were stained with cytofix/cytoperm (BD Biosciences) with mouse anti-IL-22R1 (clone 305405, R&D Systems)/mouse IgG_1_ isotype control (clone 11711, R&D Systems) or rabbit anti-IL-10R2 (sc-69580, Santa Cruz Biotechnology)/rabbit IgG isotype control (AB-105-C, R&D Systems) primary antibodies and followed by donkey anti-mouse IgG AF546 and goat anti-rabbit IgG AF488 (Invitrogen) secondary antibodies. Alternatively, cell suspension was directly used for staining as described in “Proliferation and cell death/apoptosis assays” section. Cells were processed with an LSRII flow cytometer (BD Biosciences) and were analyzed with FlowJo software (version 9.1.11, Treestar).

### PBMC isolation and cell sorting

Peripheral blood mononuclear cells (PBMC) were freshly isolated by Ficoll (GE Healthcare Biosciences) as described previously [37]. PBMC subpopulations were sorted with anti-CD4, anti-CD8, anti-CD14, anti-CD19, and anti-CD56 MicroBeads (Miltenyi Biotec) with an autoMACS Pro Separator (Miltenyi Biotec) according to manufacturer’s instructions. The purity of sorted cells was checked by flow cytometry with the following antibodies: anti-CD4 APC-H7 (clone SK3, BD Biosciences), anti-CD4 ECD (clone SFCI12T4D11, Beckman Coulter), anti-CD8 FITC (clone RPA-T8, BD Biosciences), anti-CD11c APC (clone B-ly6, BD Biosciences), anti-CD14 PB (clone M5E2, BD Biosciences), anti-CD19 PE (clone HIB19, eBioscience), and anti-CD56 PE-Cy7 (clone NCAM16.2, BD Biosciences) on a LSRII flow cytometer (BD Biosciences). We did not pursue the proposed experiments if the purity of sorted cells was less than 90 %. Analyses were performed using FlowJo software (Treestar).

### Generation of in vitro monocyte-derived dendritic cells

Sorted CD14^+^ cells were incubated for 6 days at 1*10e6 cells per ml in Roswell Park Memorial Institute (RPMI; Gibco, Life Technologies) supplemented with 10 % FCS (heat inactivated, PAA Laboratories), 50 ng/ml premium grade recombinant granulocyte macrophages colony-stimulating factor (GM-CSF) and 20 ng/ml premium grade recombinant IL-4 (Miltenyi Biotec) to obtain differentiated monocyte-derived DCs (moDCs). Cell differentiation quality was checked by flow cytometry with the following antibodies: anti-CD11c APC (clone B-ly6, BD Biosciences) and anti-CD14 PB (clone M5E2, BD Biosciences). Proper differentiation was considered as completed when at least 80 % of the harvested cells were CD11c^+^CD14^−^. For mRNA analysis, moDCs were lysed with RLT Plus buffer (Qiagen) and kept at −20 °C until further extraction.

### Leukocyte stimulation

Whole PBMC were left untreated or stimulated with 100 ng/ml staphylococcal enterotoxin B (SEB, Sigma) at 2*10e6 cells per ml for 18 h at 37 °C. Supernatants were harvested and stored at −80 °C until use. CD4^+^, CD8^+^, CD14^+^, CD19^+^, CD56^+^ sorted cells, and moDCs were either left untreated or stimulated at 2*10e6 cells per ml with 100 ng/ml SEB, 1 μg/ml resiquimod (R848) (InvivoGen)—a toll-like receptor 7 and 8 ligand, a potent activator of both monocytes and B cells—, 10 μ/ml CD3/28 beads (Gibco, Life Technologies) for 18 h or 100 ng/ml phorbol myristate acetate (PMA, Sigma) and 1 μg/ml ionomycin (iono, Sigma) for 6 h at 37 °C.

For mRNA analysis, cells were lysed with RLT Plus buffer (Qiagen) and kept at −20 °C until further extraction.

### ELISA

Measurement of IL-22 in the serum, cerebrospinal fluid (CSF), or supernatant of stimulated PBMC was performed with the Human IL-22 ELISA Ready-SET-Go (eBioscience) according to manufacturer’s instructions and read with Opsys MR (Dynex International) instrument.

IL-22BP was measured in the serum and CSF by a home-made kit. “Maxisorp Immunoplates” 96-well plates (Nunc) were coated with coating solution (15 mM Na_2_CO_3_, 34.8 mM NaHCO_3_) mixed with goat anti-IL-22BP antibody (AF1087, R&D Systems) diluted 1:500 and incubated overnight at 4 °C. The next day, blocking was performed by filling 200 μl/well PBS containing 0.05 % Tween 20 and 1 % bovine serum albumin (BSA) (PBS/T-1 % BSA) (Sigma) with 2 h incubation at 37 °C. After three washes with wash buffer solution (BD Biosciences), 100-μl standard dilutions (1087-BP-025, R&D Systems) and samples diluted with 50 μl PBS/T-1 % BSA were added to each well and incubated overnight at 4 °C. The third day, plates were washed three times and 50 μl/well of rabbit anti-IL-22BP (sc-134974, Santa Cruz Biotechnology) diluted 1:200 in PBS/T-1 % BSA were added and 1 h incubation at 37 °C was performed. Following three washes, addition of 50 μl of mouse anti-rabbit biotinylated antibody (550346, BD Biosciences) diluted 1:3,000 in PBS/T-1 % BSA was performed and the plates were incubated 1 h at 37 °C. After another round of three washes, 50 μl/well of 1:200 streptavidin-HRP (DY998, R&D Systems) in PBS/T-1 % BSA was added and incubated at RT for 30 min. Finally, after six washing steps, ELISA was revealed and developed with 100 μl/well revelation buffer (DY999 R&D Systems) in a 20-min incubation period at RT, protected from light. Reaction was stopped with 50 μl/well 1 M H_2_SO_4_, and plates were read at 450 nm with Opsys MR (Dynex International) device. Detection limit was set at 0.5 ng/ml to fully guarantee specificity and reliability of positive samples, based on data of the recombinant IL-22BP standard curve (R&D Systems).

### RNA extraction, reverse transcription and quantitative PCR

The biological material was lysed with RLT Plus buffer (Qiagen) and stored at −20 °C until RNA extraction. The RNA isolation was performed with the RNeasy Plus Mini kit (Qiagen). Up to 0.5-μg purified RNA (concentration measured with a NanoDrop 2000, Thermo Scientific) was taken for reverse transcription utilizing the Quantitect Reverse Transcription kit (Qiagen). Quantitative PCR was performed with the QuantiTect SYBR green PCR mix (Qiagen) and QuantiTect primer assays for 18S ribosomal RNA, IL-22BP set (Qiagen). MicroAmp Fast Optical 96-well reaction plate (Applied Biosystems, Life Technologies) was run with a StepOnePlus Real-Time PCR System (Applied Biosystems, Life Technologies). The relative expression of each sample was calculated with the “2e(−ΔCt)” equation where the Ct is defined as the cycle number at which the SYBR green fluorescence crosses the threshold (arbitrary set and fixed for all experiments performed) and the Δ is the difference between the Ct of the sample tested and the housekeeping gene Ct. Melting curve analysis was performed to ensure reaction specificity.

### Immunohistochemistry

For immunohistochemistry staining, tissue sections were treated with 0.6 % hydrogen peroxide in 80 % methanol for 30 min to inactivate endogenous peroxidase and blocked with blocking buffer (1 % normal donkey serum, 0.1 % Triton, 0.05 % Tween) for 1 h. The tissue sections for myelin oligodendrocyte glycoprotein (MOG) were then additionally defatted in 100 % methanol for 8 min at −20C°. Sections were incubated with following primary antibodies overnight at 4 °C: mouse anti-MOG (clone Z12, kindly provided by R. Reynolds) to target myelin, mouse anti-IL-22R1 (clone 305405, R&D Systems)/mouse IgG_1_ isotype control (clone 11711, R&D Systems), rabbit anti-glial fibrillary acidic protein (GFAP) (AB5804, Millipore; Z0334, DakoCytomation) to label astrocytes, and rabbit anti-Caveolin-1 (Cav-1) (N-20, Santa Cruz Biotechnology) for endothelia staining/rabbit IgG isotype control (AB-105-C, R&D Systems; 12–370, Millipore). Secondary biotinylated antibodies (Vector Laboratories) were applied for 1 h at room temperature, together with 4′,6-diamidino-2-phenylindole (DAPI, Invitrogen Life Technologies) counterstaining, followed by avidin-biotin complex reagent (Vector Labs) for 30 min. Color reaction was performed with 3-amino-9-ethylcarbazole. Cells were stained in hematoxylin for 5 min and rinsed afterwards under running tap water. Image acquisition was performed with a Zeiss Axiovision (Carl Zeiss Microscopy) microscope, and picture analysis was performed with Axiovision software (version V4.8.1.0, Carl Zeiss Microscopy).

### Laser scanning confocal microscopy

Immunostainings were performed with the following primary antibodies: goat anti-IL-22 (AF782, R&D Systems)/goat IgG (AB-108-C, R&D Systems), mouse anti-IL-22R1 (clone 305405, R&D Systems)/mouse IgG_1_ (clone 11711, R&D Systems), rabbit anti-IL-10R2 (sc-69580, Santa Cruz Biotechnology), rabbit anti-GFAP (AB5804, Millipore; Z0334, DakoCytomation), mouse anti-GFAP-Cy3 (for HA only, clone G-A-5, Sigma), rabbit anti-Caveolin-1 (N-20, Santa Cruz Biotechnology)/rabbit IgG (AB-105-C, R&D Systems; 12–370, Millipore), sheep anti-von Willebrand factor (VWF) (GTX74137, GeneTex) for vessel labeling, and chicken anti-microtubule-associated protein (MAP)-2 (ab5392, Abcam) to target neurons/chicken IgG (GTX35001, GeneTex) and with the following secondary antibodies: donkey anti-goat IgG AF488, donkey anti-mouse IgG AF546 and AF647, goat anti-rabbit IgG AF488, donkey anti-rabbit IgG AF647, goat anti-chicken IgG AF647, goat anti-sheep AF647 (Invitrogen) and, finally, donkey anti-rabbit IgG AF488 (Jackson ImmunoResearch) and donkey anti-rabbit IgG CSL467 (Santa Cruz Biotechnology). To reduce autofluorescence, tissue sections of the Basel cohort were incubated in cupric sulfate in ammonium buffer (10 mM CuSO_4_, 50 mM CH_3_COONH_3_, pH 5.0) for 30 min before secondary antibody staining. Nuclei staining was done with DAPI (Invitrogen Life Technologies). Slices were mounted with Vectashield (Vector Laboratories). Image acquisition was done with a Zeiss LSM 710 Quasar (Carl Zeiss Microscopy) confocal, and picture analysis was performed with Zeiss ZEN 2009 (Carl Zeiss Microscopy), ImageJ (version 1.46r, National Institutes of Health), and Axiovision softwares (version V4.8.1.0, Carl Zeiss Microscopy). Images were taken, and post-acquisition processing (brightness and contrast) was done the same way for specific antibodies and their isotype controls.

### Proliferation and cell death/apoptosis assays

For functional experiments, cells were cultured at low passage (three to six passages) in complete astrocyte medium, prior to transfer in 24-well plates (Costar) at 20,000 cells/well in RPMI only medium, 300 μl/well, on day −1. On day 0 and then every other day over a 9-day period, HA were treated with six different conditions: astrocyte medium without FCS as reference medium; RPMI only (referred as “untreated” or negative control) as standard minimal medium to provide only essential nutrient to the cells; IL-22 at 50 ng/ml (R&D Systems); tumor necrosis factor (TNF)α at 10 ng/ml (R&D Systems); TNFα and IL-22 co-treatment; and finally, 100 nM staurosporine (STS, Sigma) as positive control to induce apoptosis and cell death. TNFα was chosen as a pro-inflammatory cytokine considering its paramount role in MS [38].

To assess for cell proliferation, carboxyfluorescein succinimidyl ester (CFSE, Biolegend) staining was done at the beginning of the kinetic (day −1), such as already performed in the lab [39], whereas to assess for cell death and apoptosis, staining with 7-aminoactinomycin D (7-AAD, BD Biosciences) and Annexin V AF647 (Invitrogen Life Technologies) in Annexin-binding buffer (Invitrogen Life Technologies), respectively, were performed at day 1, 2, 3, 4, 5, 7, and 9, according to manufacturer’s instructions. Annexin V marker analysis was performed on 7-AAD^−^ pregated cells. Samples were run with an LSRII flow cytometer (BD Biosciences) and were analyzed with FlowJo software (version 9.1.11, Treestar).

### Statistical analysis

Statistical analyses were performed with GraphPad Prism software (version 6.04, GraphPad Software). The differences among groups (three or more) were first tested using Kruskal-Wallis test for multiple non-normally distributed variables. Unpaired non-parametric two-tailed Mann–Whitney was used to test groups two-by-two. A *P* value < 0.05 was considered significant.

## Results

### Increased IL-22 in active MS patients

**Fig. 1:**
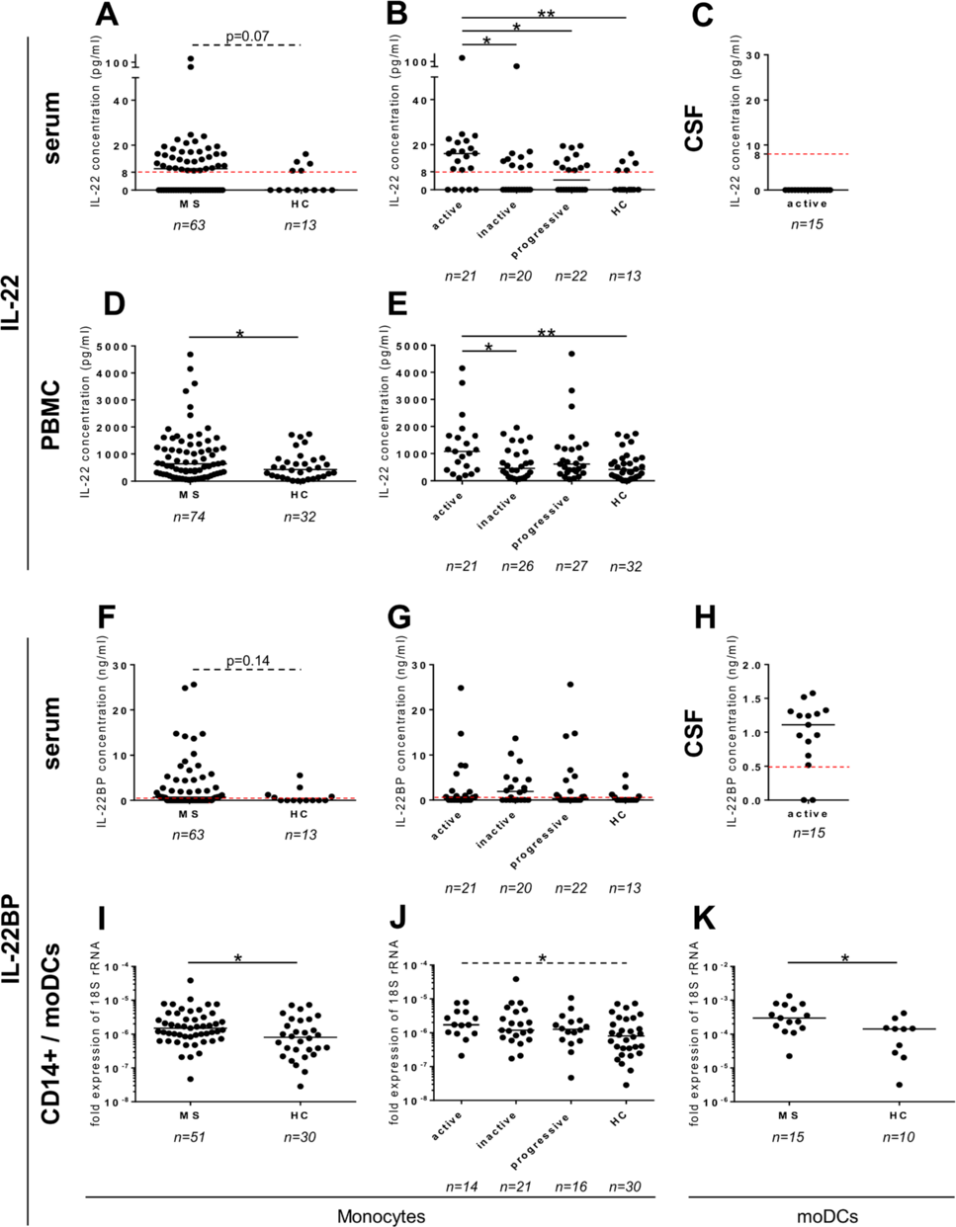
IL-22BP and IL-22 are increased in MS patients as compared to healthy controls. The IL-22 and IL-22BP expressions were assessed by ELISA (**a–h**) and qPCR (**i–k**) in the serum (**a**, **b** and **f**, **g**), CSF (**c**, **h**), isolated monocytes (**i**, **j**), and moDCs (**k**) and supernatant of SEB-stimulated PBMC for 18 h (**d**, **e**) isolated from MS patients and healthy controls. Each *dot* represents a patient. The *bars* represent the median. *Dashed red lines* represent the detection limit. *Active*: clinically active MS patients, *inactive*: clinically inactive MS patients, *progressive*: primary and secondary progressive MS patients. Differences among the four groups were significant with Kruskal-Wallis test (**b**, **e**). Unpaired non-parametric Mann–Whitney test was used to compare groups two-by-two. *P < 0.05, **P < 0.01

First, using ELISA, we found that there was a strong trend (*p* = 0.07) for an increase of IL-22 protein in the serum of 63 MS patients as compared to 13 HC (Fig. 1a). Interestingly, the level of IL-22 in the serum of MS patients with active disease was higher than in the serum of inactive (*p* = 0.017) and progressive (*p* = 0.015) MS patients and, especially, of HC (*p* = 0.003) (Fig. 1b). IL-22 was not detectable in the CSF of patients with active MS (Fig. 1c). Of note, no lumbar puncture was performed in the other categories of study patients.

Next, we found that the supernatant of SEB-stimulated PBMC of 74 MS patients secreted a higher amount of IL-22 than of 32 HC (*p* = 0.0436, Fig. 1d), a finding which was ascribable to the active category of MS patients (active versus HC: *p* = 0.0048, active versus inactive: *p* = 0.0216, Fig. 1e). Then, we investigated which leukocyte subtypes secreted IL-22. We found that CD4^+^ T cells accounted for most of the production of IL-22; nevertheless, and as already reported, monocytes, B cells, CD8^+^ T cells, and natural killer (NK) cells were also able to produce and secrete significant amount of IL-22 (Additional file 1: Figure S1) [40]. Of note, unstimulated PBMC released a low, but not null, level of IL-22, consistent with previous reports [41]. Therefore, we tested several polyclonal stimulations (SEB, R848, PMA/ionomycin, and CD3/CD28 beads), and all showed similar efficacy, except for R848 which was induced much less IL-22 secretion from CD4^+^ T cells than other stimulants (Additional file 1: Figure S1).

To further examine the putative implication of IL-22 in MS, we looked at its soluble antagonist, i.e., IL-22BP. Indeed, IL-22BP gene polymorphism has been recently associated with MS [32]. Looking first at the protein level, we did not find a difference in terms of IL-22BP protein in the sera of 63 MS patients versus 13 healthy controls (HC); however, there was a trend (*p* = 0.14) towards an increased secretion of IL-22BP in MS patients as compared to HC (Fig. 1f). Those 76 study subjects and patients were the very same who were tested for the content of IL-22 in the serum (see above). Some MS patients harbored high levels of soluble IL-22BP, reaching levels of 10 ng/ml and more (Fig. 1f–g); however, there was no difference between the categories of MS patients (Fig. 1g). Interestingly, IL-22BP was detected in the CSF of 13/15 active MS patients who had a lumbar puncture at the same time as this assay (Fig. 1h).

Then, we found that among different sorted subpopulations of blood immune cells, CD14^+^ monocytes and, especially, in vitro differentiated moDCs contained the highest levels of mRNA coding for IL-22BP (Additional file 2: Figure S2), confirming previous literature data [31, 35, 42, 43]. We found an increased expression of IL-22BP mRNA in the monocytes of 51 patients as compared to 30 HC (*p* = 0.016; Fig. 1i) and in the moDCs of a subset of 15 MS patients as compared to 10 HC (*p* = 0.016; Fig. 1k). Interestingly, although there was no difference between the categories as a whole (*p* = 0.108 with the Kruskal-Wallis test), the higher expression of IL-22BP mRNA in the monocytes of MS patients seemed to be mostly accountable to the category of active MS (active MS patients versus HC: *p* = 0.037, Fig. 1j).

### IL-22 and IL-22R1 expression in the human brain

The fact that IL-22 and its soluble antagonist receptor, IL-22BP, are differentially expressed in MS patients as compared to HC suggests that this cytokine may be involved in the immunopathogenesis of MS. For this reason, we looked for putative target cells of IL-22 in the CNS. This cytokine is recognized through the IL-22 heterodimeric receptor composed of IL-10R2 and IL-22R1 subunits [44]. Of note, IL-10R2 can also bind IL-10, IL-26, IL-28(α/β), and IL-29, whereas IL-22R1 binds also IL-20 and IL-24 [30, 45], but only IL-22 is recognized by these two subunits together [45]. IL-10R2 has been previously shown to be expressed ubiquitously among tissues from hematopoietic and nonhematopoietic origins, whereas IL-22R1 expression was absent on hematopoietic cells [14, 41].

**Fig. 2:**
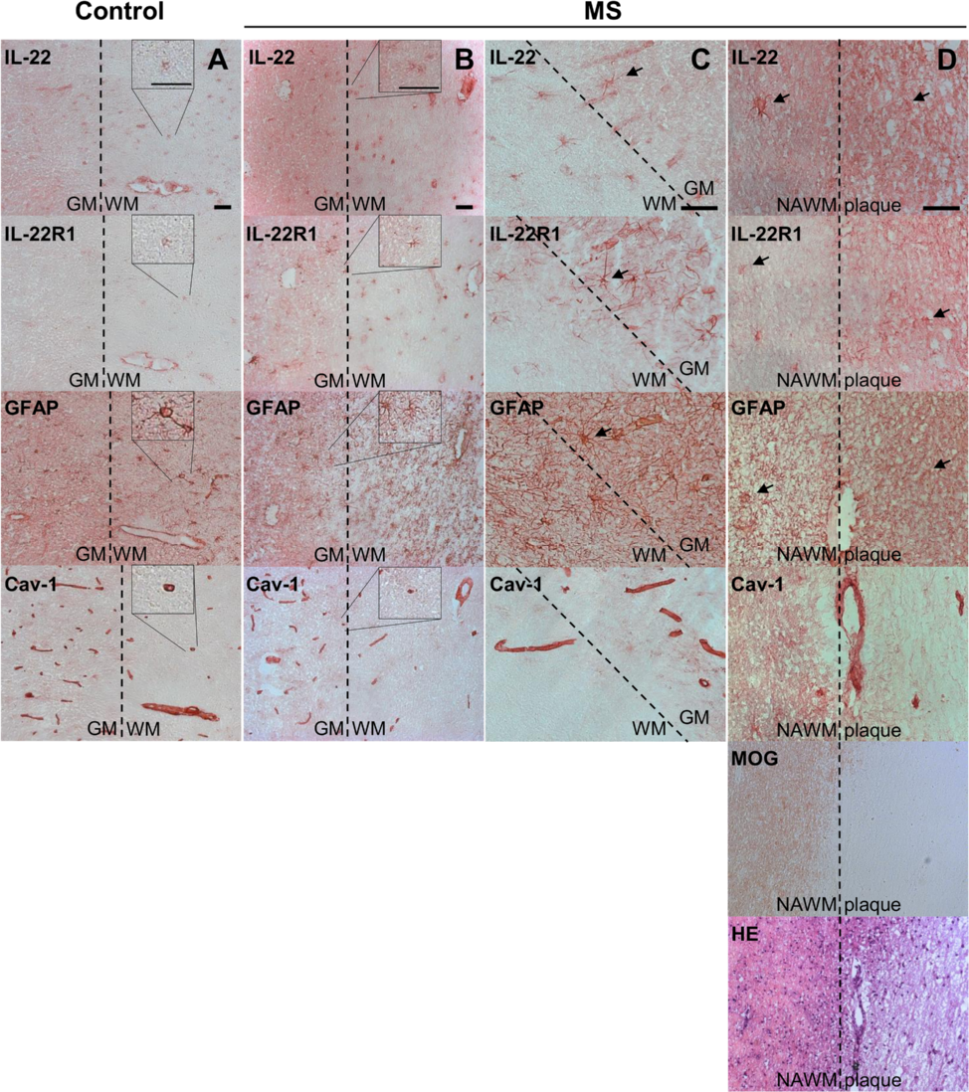
IL-22 and IL-22R1 are expressed in human brain. IL-22, IL-22R1, GFAP, and Caveolin-1 immunohistochemistry peroxidase stainings of brain tissue sections of control (**a**) and MS (**b**–**d**) patients. MOG and HE stainings were used to detect MS demyelinating plaques (**d**). All four pictures belonging to one column (**a**, **b**, **c**, or **d**) were always immediately adjacent to each other. Pictures **a**, **b**, and **c** were taken at areas at the border between GM and NAWM, whereas pictures in **d** were taken from the same location at the edge between NAWM and a plaque. Inserts in columns **a** and **b** represent a threefold magnification of the selected area. *Arrow*: astrocyte-like pattern. **a** study patient B-C2, **b** and **d** study patient B-MS3, **c** study patient B-MS5 (Table 2). *Scale bar*, 50 μm (**a**, **b**: ×20, **c**, **d** ×40). *GM*: gray matter, *NAWM*: normal appearing white matter, *WM*: white matter. Representative pictures obtained from the observations of seven control and five MS autopsy samples

Here, to explore whether human brain CNS cells express the IL-22 receptor and whether IL-22 is present in the brain, we analyzed brain tissue sections from five MS cases and 18 non-MS controls. Using immunohistochemistry peroxidase staining, we aimed at detecting IL-22 and IL-22R1 on adjacent tissue sections with pictures taken at the exact same area for all samples (Fig. 2). We were able to detect IL-22 and IL-22R1 in the GM and the WM of both control subjects (Fig. 2a) and MS patients, either in areas deprived of lesions (Fig. 2b, c) or at the vicinity of plaques (Fig. 2d). Isotype controls showed that there was no unspecific staining neither of the primary nor of the secondary antibodies used in Fig. 2 (Additional file 3: Figure S3). Of note, MS plaques were identified by MOG and HE staining (example is shown in Fig. 2d). The expression of the cytokine and its receptor was clearly stronger in MS than control tissue (Fig. 2b–d compared to Fig. 2a). Interestingly, the morphology of the IL-22- and IL-22R1-positive cells clearly looked alike GFAP-positive astrocytes (Fig. 2c, arrow, first to third row), suggesting that the receptor and its cytokine were present on the same cells, for example see the two upper panels of Fig. 2c. The IL-22- and IL-22R1 expressing cells were often found in the vicinity of blood vessels. Since IL-22R1 was identified to be expressed by endothelial cells in MS [22], we stained for Caveolin-1. However, we found that the staining of IL-22 and its receptor reflected more the staining of GFAP than the one of Caveolin-1 (Fig. 2c, d in particular). Caveolin-1 delineates very nicely in blood vessels, whereas IL-22 and IL-22R1 are localized on fine processes around blood vessels and on cell bodies of stellate cells in proximity of blood vessels. Moreover, in MS lesions where strong astrogliosis occurs, IL-22 and IL-22R1 expressions was also enhanced (Fig. 2d).

**Fig. 3:**
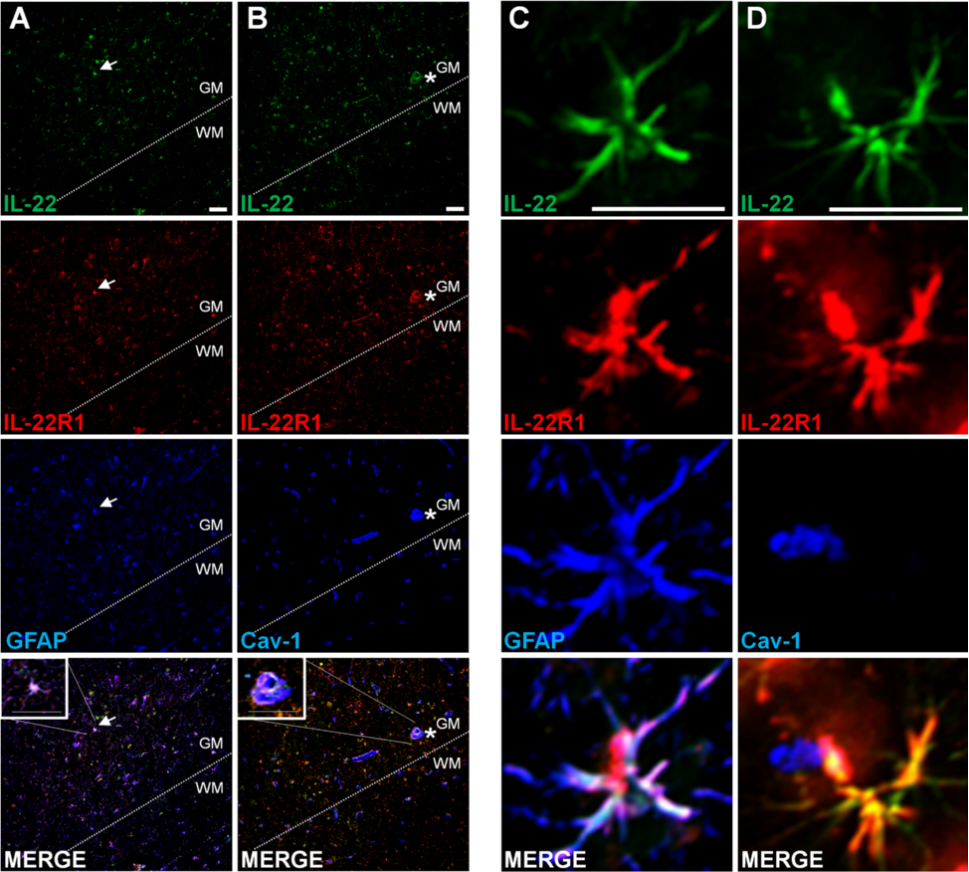
Specific expression of IL-22 and its receptor, IL-22R1, by astrocytes in the brains of control patients without MS and suffering from other neurological diseases. Laser scanning confocal microscopy observations were performed in brain tissue autopsies (Basel cohort). Brain autopsy labeled for IL-22 (*first panel*, *green*), IL-22R1 (*second panel*, *red*), and Caveolin-1 or GFAP (*third panel*, *blue*). Merged images are depicted as composite images in the lower panel (colocalization of IL-22R1 and GFAP appears in *yellow* and triple colocalization of IL-22, IL-22R1, and GFAP in *white*). Inserts are a ×10 zoom of the selected area (**a**, **b**, *lowest panels*). Images **a** and **b** were taken on autopsied brain tissue from control patient B-C6 and **c** and **d** from control patient B-C1 (Table 2). *Arrows* in **a** point at astrocytes, and *stars* in **b** at blood vessels. *Bars*, 50 μm. *GM*: gray matter, *WM*: white matter. Representative pictures obtained from the observations of seven control autopsy samples

**Fig. 4:**
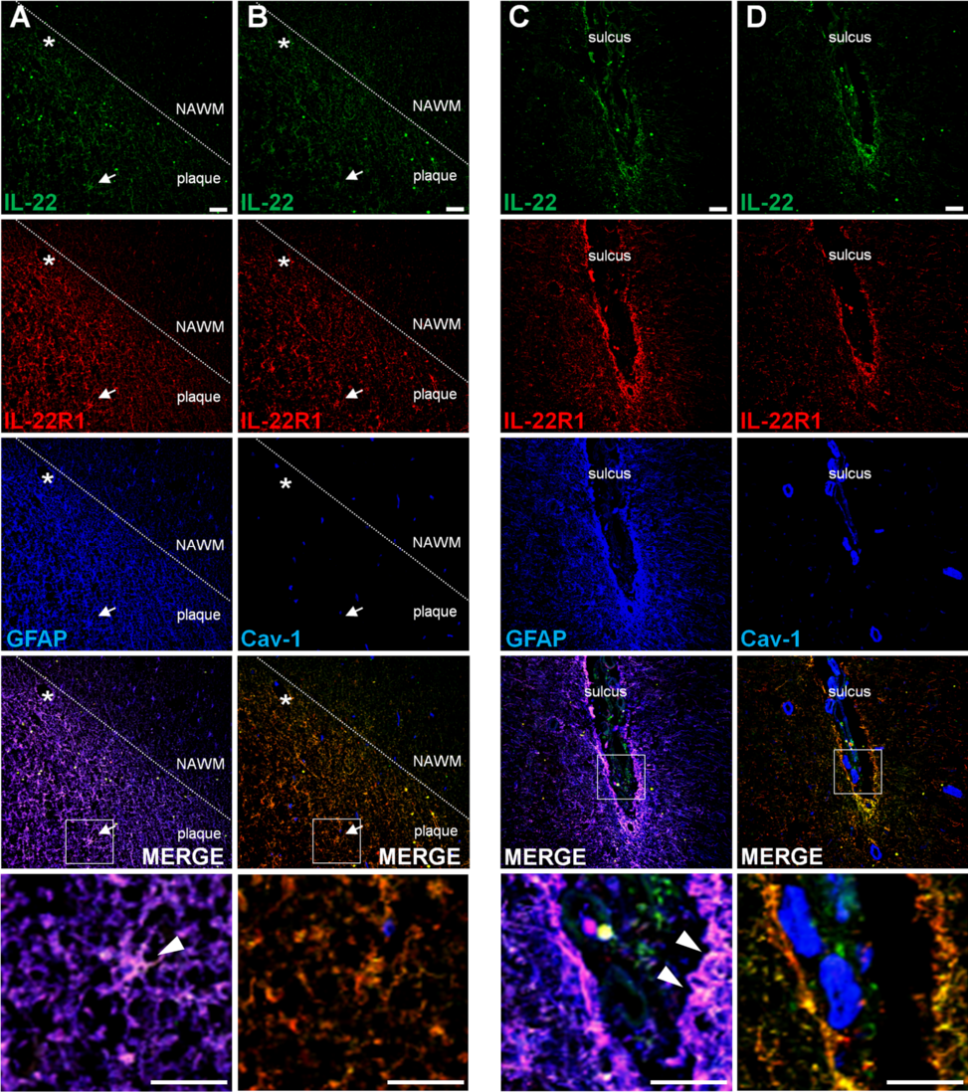
Strong expression of IL-22 and its receptor, IL-22R1, in the plaques of MS brains. Immunofluorescence of human brain tissue sections stained for IL-22 (*green*), IL-22R1 (*red*), and Cav-1 or GFAP (*blue*) in MS brain tissue autopsies. Images were taken from patient B-MS3 patient (Table 2) in a plaque located in the subcortical WM (**a**, **b**) and in a plaque located in the subpial GM (**c**, **d**). The lowest panels depict a ×5 magnification of the white squares displayed in the above panels. *Arrow*: astrocytes, *star*: blood vessels. *Arrowheads* point at triple IL-22/IL-22R1/GFAP colocalization (**a**, **c**). *Bars*, 50 μm. *NAWM*: normal appearing white matter. Representative pictures obtained from the observations of five MS autopsy samples

Next, using immunofluorescent confocal microscopy, we wanted to determine whether IL-22 and IL-22 receptor indeed colocalize on the same cells. We also wanted to ascertain their expression on astrocytes as indicated by light microscopy (Fig. 2). We found that IL-22 colocalized with its receptor in the brain tissues of control subjects (Fig. 3a–d) as well as in the brain tissues of MS patients (Fig. 4). In the brain of control subjects, the IL-22/IL-22R1 colocalization seemed to be slightly more expressed in the GM than in the WM (Fig. 3a, b), whereas in MS, it was clearly stronger in the plaques, either in the WM (Fig. 4a, b) or the subpial GM (Fig. 4c, d), than in the normal appearing white matter (NAWM) (Fig. 4a, b). We were also able to confirm that the IL-22/IL-22R1 couple was expressed on astrocytes in the brain of control subjects (Fig. 3a, c) as well as of MS patients (Fig. 4a, c). By contrast, co-staining of the IL-22/IL-22R1 with Cav-1 was rarely detected (less than 1 % of Cav-1^+^ structures were also positive for IL-22/IL-22R1) (Fig. 3b). By analyzing adjacent slices of pictures taken at the very same location, we noticed strong IL-22R1 expression in white matter plaques (Fig. 4a, b) as well as in subpial lesions in gray matter (Fig. 4c, d).

To ascertain the specificity of the antibodies used for immunofluorescent confocal microscopy and to rule out autofluorescence, we performed isotype stainings, using the exact same protocol, acquisition parameters, and post-processing analysis methodology as for specific antibodies (Additional file 4: Figure S4 and Additional file 5: Figure S5). Of note, the pictures of the isotype controls were taken at the very same location in the tissue slices as the pictures of the specific antibodies, except for Additional file 4: Figure S4c, which was not immediately adjacent to Fig. 3c, d. Otherwise, tissue pictured in Additional file 4: Figure S4a, b was immediately adjacent to the one represented in Fig. 3a, b; the same was true for Additional file 5: Figure S5a with Fig. 4a, b and Additional file 5: Figure S5b with Fig. 4c, d.

Additional staining performed on biopsies from control patient L-C5 confirmed that there was a very strong colocalization of IL-22R1 with GFAP (Additional file 6: Figure S6a) but not with Cav-1 (Additional file 6: Figure S6b, c) or with MAP-2 (Additional file 6: Figure S6c). Staining with anti-VWF, an alternative blood vessel marker, led to the same results as with anti-Cav-1, i.e., no colocalization with IL-22R1 (Additional file 7: Figure S7). Interestingly, IL-22R1 expression was particularly high in the close vicinity of blood vessels, lining them up. However, we could show that this proximity was not due to endothelial expression of IL-22R1 (Additional file 6: Figure S6b) but was rather attributable to expression by astrocytic feet. Of note, to ascertain that the detection of IL-22R1 used so far indeed indicated the expression of the whole IL-22 receptor, we assessed the expression of the other subunit of the IL-22 receptor, i.e., IL-10R2, and found that, indeed, IL10R2 colocalized with IL-22R1, clearly establishing that the heterodimeric IL-22 receptor complex is fully expressed in the CNS (Additional file 6: Figure S6d). Altogether, this set of experiments establishes that the IL-22 receptor is expressed in the human brain, mostly on astrocytes, and that IL-22 colocalize with its receptor. We also demonstrate that even if the IL-22/IL-22R1 couple is present in the brain of control patients with other neurological disease than MS, it nevertheless predominates in the brain of MS patients, in particular in the plaques.

### IL-22 increased the survival of astrocytes

**Fig. 5:**
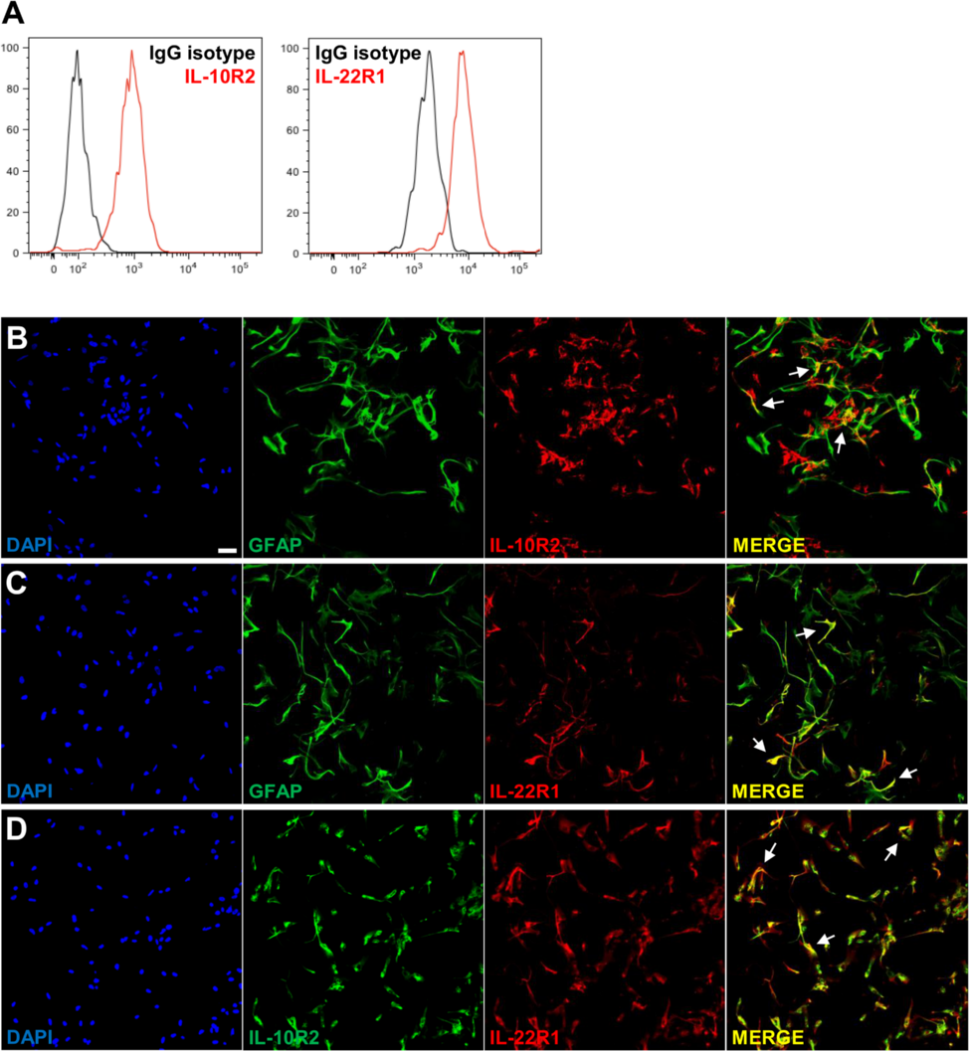
Colocalization of both subunits of IL-22 receptor on primary astrocytes. **a** Flow cytometry experiments show the expression of both subunits of the IL-22 receptor, IL-10R2, and IL-22R1 (in *red*), on human primary astrocytes (HA) as compared to their isotype control counterparts (in *black*). **b**–**d** By immunofluorescent confocal microscopy, there is a colocalization of GFAP (*green*) with IL-10R2 (*red*) (**b**) and with IL-22R1 (*red*) (**c**) as well as of IL-10R2 (*green*) with IL-22R1 (*red*) (**d**). The colocalization appears is depicted in *yellow* (*arrows*, *MERGE*). DAPI is represented in *blue* and not included in MERGE overlay. *Bars*, 50 μm

Having shown that the IL-22 receptor was expressed in the human brain, predominantly by astrocytes, we decided to investigate the role of IL-22 on astrocytes. To this end, we selected human primary astrocytes. The purity of those primary astrocytes, as assessed by GFAP staining on flow cytometry, was close to 80 % (Additional file 8: Figure S8). Such as shown by flow cytometry (Fig. 5a) and by immunofluorescence (Fig. 5b–d), these astrocytes expressed both subunits of the IL-22 receptor, confirming that IL-22 could bind to these cells.

In order to determine whether IL-22 has a protective or deleterious effect on astrocytes, we studied its impact in terms of cell death, such as measured by 7-AAD, a marker of cell viability.

**Fig. 6:**
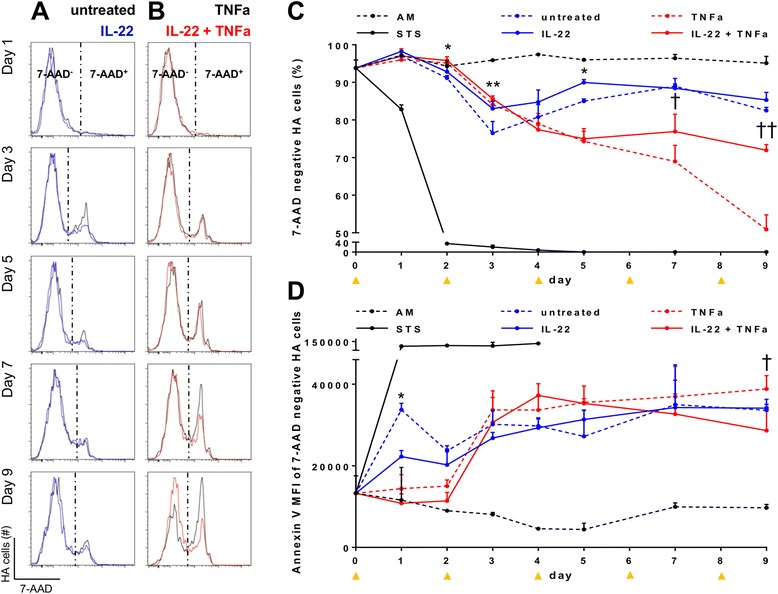
Enhanced survival of IL-22-treated primary astrocytes in insulting conditions. Primary astrocyte cells (*HA*) were cultured in 24-well plates starting at day −1 and were treated every second day starting at day 0 with astrocyte medium (*AM*)—representing the optimal culture medium for astrocytes—or with RPMI (poor medium, hereafter referred to as *untreated*), with or without addition of the following: IL-22 alone, TNFα alone, IL-22 and TNFα together, or still staurosporine (*STS*) alone, the latter representing a potent inducer of apoptosis. Cells were stained with 7-AAD and Annexin V and analyzed by flow cytometry to assess their survival and apoptotic status. *Histograms* represent 7-AAD profile of untreated versus IL-22 (**a**) and of TNFα versus IL-22 + TNFα (**b**). Survival profile analysis was performed by flow cytometry by following the frequency of living cells (i.e., 7-AAD-negative cells, Fig. 6 **c**) and, among those that were not dead (7-AAD-negative cells), by following the proportion of those surviving cells but which underwent apoptosis (**d**). Each *dot* represents the median of six replicates, except for AM and STS conditions (three replicates). *Orange arrows* indicate treatment renewal. Considering STS treatment, 7-AAD kinetic was stopped after 4 days as all recovered cells were dead at this time point.*comparison of IL-22-treated versus untreated cells; †comparison of TNFα- versus TNFα + IL-22-treated cells. The *vertical bars* determine the 75th percentile of the median. Significance was calculated with unpaired non-parametric Mann–Whitney test. * or †*P* < 0.05, ** or ††*P* < 0.01

We found that IL-22 protected human astrocyte from cell death. Indeed, nutriment-deprived astrocytes that had been treated with IL-22 (plain blue line) exhibited a significant higher survival than astrocytes cultured in the same conditions but without IL-22 (dashed blue line) (Fig. 6a, c). Even if the improved survival of astrocytes attributable to IL-22 was relatively mild (increase of survival 2.4 % on day 2, 7 % on day 3, and 4.2 % on day 5), it was significant and occurred during a time of the culture (day 2, 3, and 5), which corresponded to a nadir in terms of astrocyte survival (Fig. 6c, blue lines).

Then, we assessed whether the protective function of IL-22 on astrocytes was maintained in a still more hostile environment, i.e., under inflammatory conditions. Therefore, we compared TNFα-treated astrocytes with or without adding IL-22. We found that whereas there was a steady and constant cell death of the TNFα-treated astrocytes population which had not received IL-22 (dashed red line) (Fig. 6b, c), there was a much better survival of astrocytes co-treated with TNFα and IL-22 (plain red line) from day 7 (Δ = 8.9 %) to day 9 (Δ = 20.6 %) (Fig. 6b, c). Regarding TNFα- versus IL-22- and TNFα-treated human astrocyte conditions, TNFα-treated cells showed steady shrinkage of surviving cell number till the end of the kinetic (Fig. 6c). Addition of IL-22 to the TNFα-treated cells drastically ameliorated cell survival from day 7 on, as reflected by the decreased frequency of 7-AAD-positive coupled with a rise of 7-AAD-negative cell frequency, i.e., more alive astrocytes.

In an attempt to determine if an anti-apoptotic mechanism may account for the protective effect of IL-22, we assessed whether, among 7-AAD-negative living cells, there were cells in an early stage of apoptosis, such as revealed by Annexin V staining. Gating on live cells only (7-AAD-negative), we found that the mean fluorescence intensity (MFI) of Annexin V was significantly less intense in IL-22-treated cells than their untreated counterparts on the first day of culture (27.1 % MFI difference), showing that the former was less apoptotic than the latter (Fig. 6d). Furthermore, we found that IL-22 treatment significantly attenuated the pro-apoptotic effect of TNFα on day 9 of the assay (22.8 % MFI decrease, Fig. 6d).

Finally, some authors have shown that IL-22 promotes proliferation of epithelial cells [19]. Thus, we examined whether the pro-survival effect of IL-22 on astrocytes could be ascribed to this property of IL-22. However, we did not find such an effect of IL-22 (data not shown).

## Discussion

So far, IL-22 has been barely studied in the context of MS. Possible reasons may include the unchanged course of EAE in mice deficient for IL-22 as compared to wild-type mice [46] and also the fact that this cytokine does not target immune cells [14, 21, 41]. Yet, there are some elements suggesting that this cytokine may be involved in the immunopathogenesis of MS. Indeed, a polymorphism of the gene coding for interleukin-22 binding protein between MS patients and controls has been described recently [32]. Interestingly, in EAE, IL-22BP knock-out mice have a decreased neuroinflammatory profile and an overall less severe disease course as compared to wild-type littermates, strongly suggesting that IL-22 attenuates disease severity [35].

We found that the level of IL-22 was higher in the serum of MS patients than HC (Fig. 1a). In fact, this increase was entirely attributable to MS patients with active disease (Fig. 1b). This increase of serum IL-22 seems to be attributable to an increased secretion of this cytokine by PBMC (Fig. 1d, e), in particular CD4^+^ T cells (Additional file 1: Figure S1) [41]. Similarly to us, others recently found that CD4^+^ T cells, and more specifically Th17 and Th22 cells, of MS and neuromyelitis optica (NMO) patients secreted more IL-22 than those of HC [47, 48]. Our results are supported by findings in Lewis rats where the expression of IL-22 is increased during the acute phase of EAE and decreased in its recovery phase, while IL-10 and IL-17 levels remain unchanged. These variations suggest that there is a tight correlation between this cytokine and the disease course [49]. Thus, in an attempt to understand the regulation of IL-22, we examined IL-22BP, the soluble antagonist receptor of IL-22. Contrasting with IL-22, we saw no difference between MS patients and HC at the protein level. However, we found that mRNA coding for IL-22BP was mainly produced by monocytes and moDCs (Additional file 2: Figure S2), corroborating what has recently been described in mice and humans [35, 43]. We then found that the level of IL-22BP coding mRNA was higher in the monocytes and moDCs of MS patients than HC (Fig. 1i, k). Nevertheless, contrasting again with IL-22, this increase was not clearly ascribable to MS patients with active disease, even if there was a trend (Fig. 1j). Altogether, these data suggest that IL-22 is under tight control of IL-22BP, such as it is the case for instance for the control of IL-1β by IL-1RA [50]. Yet, during a relapse, IL-22 seems to “overrule” this control, such as revealed by the significant increase of IL-22 in the serum of those patients.

Somewhat contrasting with the data in peripheral blood, we found that, in the CSF of active MS patients, IL-22BP (Fig. 1h), but not IL-22 (Fig. 1c), was detectable. The absence of IL-22 in the CSF of active MS patients (Fig. 1c) may simply indicate that IL-22 plays no role at the CNS level. However, we do not think that this observation should lead to such conclusion. First, it is well established that the absence of a cytokine in the CSF does not preclude a paramount role in CNS inflammation, such as reflected, for instance, by IL-6 [32, 51]. Second, and more important, since IL-22BP was present in the CSF of active MS patients (Fig. 1h), it is tempting to hypothesize that it is in reaction to its ligand, i.e., IL-22. Third, confirming the results of others [14], we were not able to detect IL-22R1 on hematopoietic cells (data not shown), further pointing to the rationale to search for other target cells, in this case, the brain.

Thus, we examined whether brain cells did express IL-22 receptor. Whereas IL-10R2 is more or less ubiquitous [14], the expression of IL-22R1 is much more restricted. Therefore, having shown that both subunits of IL-22 receptor colocalized on astrocytes (Additional file 6: Figure S6d), we thereafter focused on the more restrictive subunit IL-22R1. A major finding was the expression of IL-22R1 on astrocytes of both control and MS patients but clearly predominating in the latter. In particular, this expression was high in MS plaques or adjacent to blood vessels, pointing to a wide expression in perivascular astrocyte end feet (Fig. 4). Such as Kebir et al., we observed colocalization of IL-22R1^+^ and caveolin-1^+^, indicating an expression of this receptor by endothelial cells [22]; but in our hands, this expression was restricted to the brain of non-MS patients and in the NAWM of one MS patient and was of limited extent. We could also rule out a constitutive expression of IL-22R1 by neurons since we never observed MAP-2^+^ and IL-22R1^+^ colocalization (Additional file 6: Figure S6c).

The IL-22 cytokine was shown to be expressed in the brain and spinal cord of mice [52, 53]. Having demonstrated that human astrocytes expressed high levels of the IL-22 receptor, we subsequently looked for IL-22 presence in the CNS. We found that this cytokine was indeed present and that it colocalized with IL-22 receptor on astrocytes, further suggesting that IL-22 targets astrocytes. However, whether the detected IL-22 was of lymphocytic origin or whether it was produced by resident cells of the CNS, for instance astrocytes themselves in an autocrine fashion, remains to be determined. The fact that IL-22 was not detected in the CSF somewhat argues for a production by CNS resident cells rather than a secretion by T cells. Nevertheless, one should not forget that IL-22 in the CSF may also be trapped by IL-22BP, since, in our hands, the latter was detected in the CSF.

Astrocytes are crucial to maintain CNS homeostasis and are now recognized to play a role in the pathogenesis of autoimmune demyelinating diseases. In NMO, a CNS disease sharing many features with MS, astrocytes play a central role as they are the cells expressing aquaporin-4 (AQP4), a water channel embodying the antigen against which the autoimmunity of the NMO antibodies is directed [54, 55]. In MS, astrocytes have been increasingly recognized as being an important component of the pathogenesis of the disease [56, 57]. KIR-4.1, which was recently found to be a possible target of auto-antibodies in human MS, is also expressed by oligodendrocytes and astrocytes [58]. To explore what could be the effect of IL-22 on astrocytes, we resorted to primary human astrocytes. We could confirm that these cells had astrocyte-characteristics and harbored both IL-22 receptor subunits (Fig. 5). We found that IL-22-treated astrocytes exhibited an increased survival as compared to untreated ones, and, interestingly, this effect was maintained in inflammatory conditions since IL-22 mitigated the effect of TNFα. This effect was mediated, at least in part, by a decrease of apoptosis. Supporting these findings, previous studies have shown that IL-22-treated rat pheochromocytoma cells exhibit a modest increased survival in serum-deprived conditions [59]. Some authors have found that IL-22 increased the proliferative function of keratinocytes [19, 60] or colonic epithelial cells [42]. While we could reproduce these data on HaCaT keratinocyte cell line, we did not observe any proliferative effect of IL-22 on primary human astrocytes (data not shown), further pointing to a pro-survival effect of IL-22 on existing astrocytes. Further studies investigating by which mechanism(s) IL-22 modulates astrocyte survival are warranted to better understand the role of this cytokine on its newly defined CNS target.

## Conclusions

In conclusion, we have shown (i) that MS patients in relapse harbor significantly higher serum levels of IL-22, (ii) that astrocytes express the IL-22 receptor, (iii) that there is a colocalization of IL-22 with astrocytes, and (iv) that IL-22 has pro-survival properties on primary human astrocytes. Astrocytes can play a dual role when challenged, depending on the nature of the insult, harboring either a beneficial or a detrimental phenotype [57]. Thus, having shown that IL-22 is a player in the immunopathogenesis of MS, we will now examine whether the net effect of IL-22 on astrocytes is pro- or anti-inflammatory.

## Additional files

Additional file 1: Figure S1. Different leukocyte subtypes are able to produce and release IL-22 upon stimulation. Total PBMC were isolated and MACS-sorted into monocytes (CD14+), B cells (CD19+), CD4^+^ T cells, CD8^+^ T cells, and NK cells (CD56+). Cells were either treated with SEB, R848, or CD3/CD28 beads (CD3/28) for 18 h, PMA/ionomycin (PMA/iono) for 6 h, or left to rest for 18 h (unstim). Upon stimulation, all leukocytes were able to secrete significant amount of IL-22. Nevertheless, CD4+ T cells represented the major IL-22 source. Except for R848, all polyclonal stimulations induced similar level of secreted IL-22 in each respective cell subpopulation. Boxes represent the median, and bars the 75th percentile. *n* = 5 study subjects. Additional file 2: Figure S2. Monocytes and moDCs are the main IL-22BP-expressing cells. Total PBMC were isolated and MACS-sorted into monocytes (CD14+), B cells (CD19+), CD4^+^ T cells, CD8^+^ T cells, NK cells (CD56+), and moDCs. Cells were either directly processed or treated for 6 h with PMA/ionomycin (PMA/iono) or stimulated for 18 h with SEB after sorting. Expression level is relative to 18S ribosomal RNA housekeeping gene. Additional file 3: Figure S3. Peroxidase stainings are specific for IL-22, IL-22R1, GFAP, and Caveolin-1 in autoptic brain tissue from control and MS subjects. Immunohistochemistry peroxidase stainings of isotype controls of goat anti-IL-22, mouse anti-IL-22R1, rabbit anti-GFAP, and rabbit anti-Cav-1 antibodies. Pictures of A, B, C, and D have been acquired at the exact same location as for specific antibodies and mirror exactly panels A, B, C, and D, respectively of Fig. 2. Protocol and images were processed exactly the same way for specific antibodies as for isotype controls. Background is negative for goat and mouse isotype controls (respectively IL-22 and IL-22R1 in Fig. 2). Background for the rabbit isotype control was somehow higher, especially in blood vessels, but remained unspecific and thus did not compromise GFAP and Cavolin-1 specificity. A: study patient B-C2, B and D: study patient B-MS3, C: study patient B-MS5 (Table 2). Scale bar, 50 μm (A–B, ×20; C–D, ×40). GM: gray matter, NAWM: normal appearing white matter, WM: white matter. Representative pictures obtained from the observations of seven control and five MS autopsy samples. Additional file 4: Figure S4. Immunofluorescence stainings are specific for IL-22, IL-22R1, GFAP and Caveolin-1 in autoptic brain tissue from control patients. Immunofluorescence staining of isotype controls of goat anti-IL-22, mouse anti-IL-22R1, rabbit anti-GFAP and rabbit anti-Cav-1 antibodies. Panel A for A, B for B, and C for C and D, respectively, are duplicates of images of Fig. 3 representing the same tissue location in the brain. Few unspecific background/autofluorescence could be observed, likely due to tissue quality (brain autopsies). The settings of isotype control experiments were exactly the same as for specific antibodies experiments (acquisition parameters and post-processing analyses). Images from panel A and B were taken from B–C6 and C from B–C1 control patient autopsy brain tissues, as for Fig. 3 (Table 2). Bars, 50 μm. GM: gray matter, WM: white matter. Representative pictures obtained from the observations of seven control MS autopsy samples. Additional file 5: Figure S5. Immunofluorescence stainings are specific for IL-22, IL-22R1, GFAP and Caveolin-1 in autoptic brain tissue from MS patients. Immunofluorescence staining of isotype controls of goat anti-IL-22, mouse anti-IL-22R1, rabbit anti-GFAP, and rabbit anti-Cav-1 antibodies. Panel A for A and B, and B for C and D, respectively, mirror exactly images in Fig. 4, being from the exact same location and processed identically (same acquisition parameters and post-processing analyses). Thin unspecific autofluorescent area in 488 and 564 nm could be observed. Pictures were taken in patient B-MS3 brain section, again, as for Fig. 4 (Table 2). Bars, 50 μm. GM: gray matter, NAWM: normal appearing white matter, WM: white matter. Representative pictures obtained from the observations of five MS autopsy samples. Additional file 6: Figure S6. Colocalization of GFAP and IL-10R2, but not Cav-1 and MAP-2 with IL-22R1 in the brain. Laser scanning confocal microscopy of brain biopsies labeled for IL-22R1 (red) in association with: A) the astrocytic marker, GFAP (green), B) the caveolin-1 endothelial marker (green), C) the caveolin-1 endothelial marker (white) and the neuronal marker, MAP-2 (green), and D) the IL-10R1 subunit receptor marker (green). DAPI staining (blue) is represented in inserts on the upper left side. Images were taken on biopsied brain tissue from control patient L-C5 (A to C) and L-C7 (D) (Table 2). Bar, 50 μm. Representative pictures obtained from the observations of 11 control biopsy samples. Additional file 7: Figure S7. There is a high degree of colocalization between von Willebrand factor and Caveolin-1, but not between IL-22R1 and any of these two endothelial cell markers in the human brain. Immunofluorescence confocal microscopy images of VWF (blue), IL-22R1 (red), and Cav-1 (green) of two slides, taken at different parts of the brain, of L-C5 study subject (Table 2) are depicted (A and B). Counterstaining was performed with DAPI. Bars, 50 μm. Additional file 8: Figure S8. Large majority of GFAP-positive cells in HA primary astrocytes. Flow cytometry histogram depicting GFAP stained HA primary astrocytes (the bar indicates GFAP-positive cells, in percent).

## Abbreviations

7-AAD: 7-aminoactinomycin D
AM: astrocyte medium
AQP4: aquaporin-4
BSA: bovine serum albumin
Cav-1: caveolin-1
CFS: cerebrospinal fluid
CFSE: carboxyfluorescein succinimidyl ester
CIS: clinically isolated syndrome
CNS: central nervous system
DAPI: 4′,6-diamidino-2-phenylindole
EAE: experimental autoimmune encephalomyelitis
FCS: fetal calf serum
GFAP: glial fibrillary acidic protein
GM: gray matter
GM-CSF: granulocyte macrophages colony-stimulating factor
HA: human astrocytes
HC: healthy control
HE: hematoxylin and eosin
IL: interleukin
IL-22BP: interleukin-22 binding protein
Iono: ionomycin
MAP-2: microtubule-associated protein 2
MCAM: melanoma cell adhesion molecule
MFI: mean fluorescence intensity
moDCs: monocyte-derived dendritic cells
MOG: myelin oligodendrocyte glycoprotein
MS: multiple sclerosis
NAWM: normal appearing white matter
NK cell: natural killer cell
NMO: neuromyelitis optica
PBMC: peripheral blood mononuclear cells
PBS: phosphate-buffered saline
PMA: phorbol myristate acetate
PP-MS: primary progressive multiple sclerosis
R848: resiquimod
RR-MS: relapsing remitting multiple sclerosis
RT: room temperature
SEB: staphylococcal enterotoxin B
SLE: systemic lupus erythomatosus
SP-MS: secondary progressive multiple sclerosis
Th cell: T helper cell
TNF: tumor necrosis factor
VWF: von Willebrand factor
WM: white matter
