# Supplementary figures and images for: Interleukin-22 is increased in multiple sclerosis patients and targets astrocytes
Source: J Neuroinflammation. 2015 Jun 16;12:119. doi: 10.1186/s12974-015-0335-3 (PMC4480507; doi:10.1186/s12974-015-0335-3)

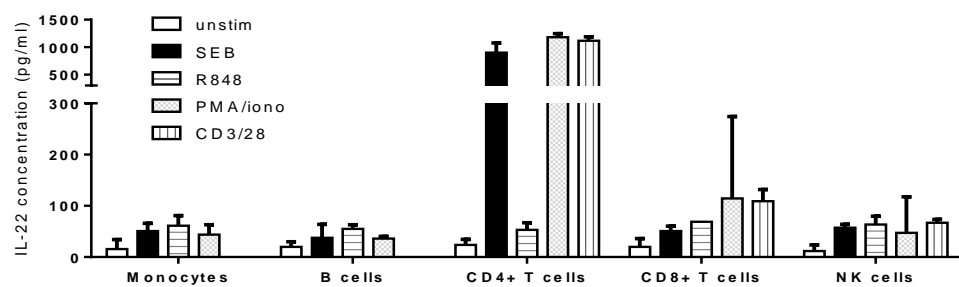

Supplement: Additional file 1: Figure S1. — Different leukocyte subtypes are able to produce and release IL-22 upon stimulation. Total PBMC were isolated and MACS-sorted into monocytes (CD14+), B cells (CD19+), CD4+ T cells, CD8+ T cells, and NK cells (CD56+). Cells were either treated with SEB, R848, or CD3/CD28 beads (CD3/28) for 18 h, PMA/ionomycin (PMA/iono) for 6 h, or left to rest for 18 h (unstim). Upon stimulation, all leukocytes were able to secrete significant amount of IL-22. Nevertheless, CD4+ T cells represented the major IL-22 source. Except for R848, all polyclonal stimulations induced similar level of secreted IL-22 in each respective cell subpopulation. Boxes represent the median, and bars the 75th percentile. n = 5 study subjects. [file 12974_2015_335_MOESM1_ESM.pdf]

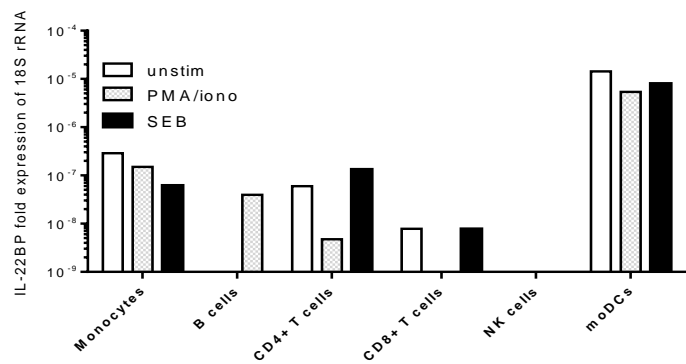

Supplement: Additional file 2: Figure S2. — Monocytes and moDCs are the main IL-22BP-expressing cells. Total PBMC were isolated and MACS-sorted into monocytes (CD14+), B cells (CD19+), CD4+ T cells, CD8+ T cells, NK cells (CD56+), and moDCs. Cells were either directly processed or treated for 6 h with PMA/ionomycin (PMA/iono) or stimulated for 18 h with SEB after sorting. Expression level is relative to 18S ribosomal RNA housekeeping gene. [file 12974_2015_335_MOESM2_ESM.pdf]

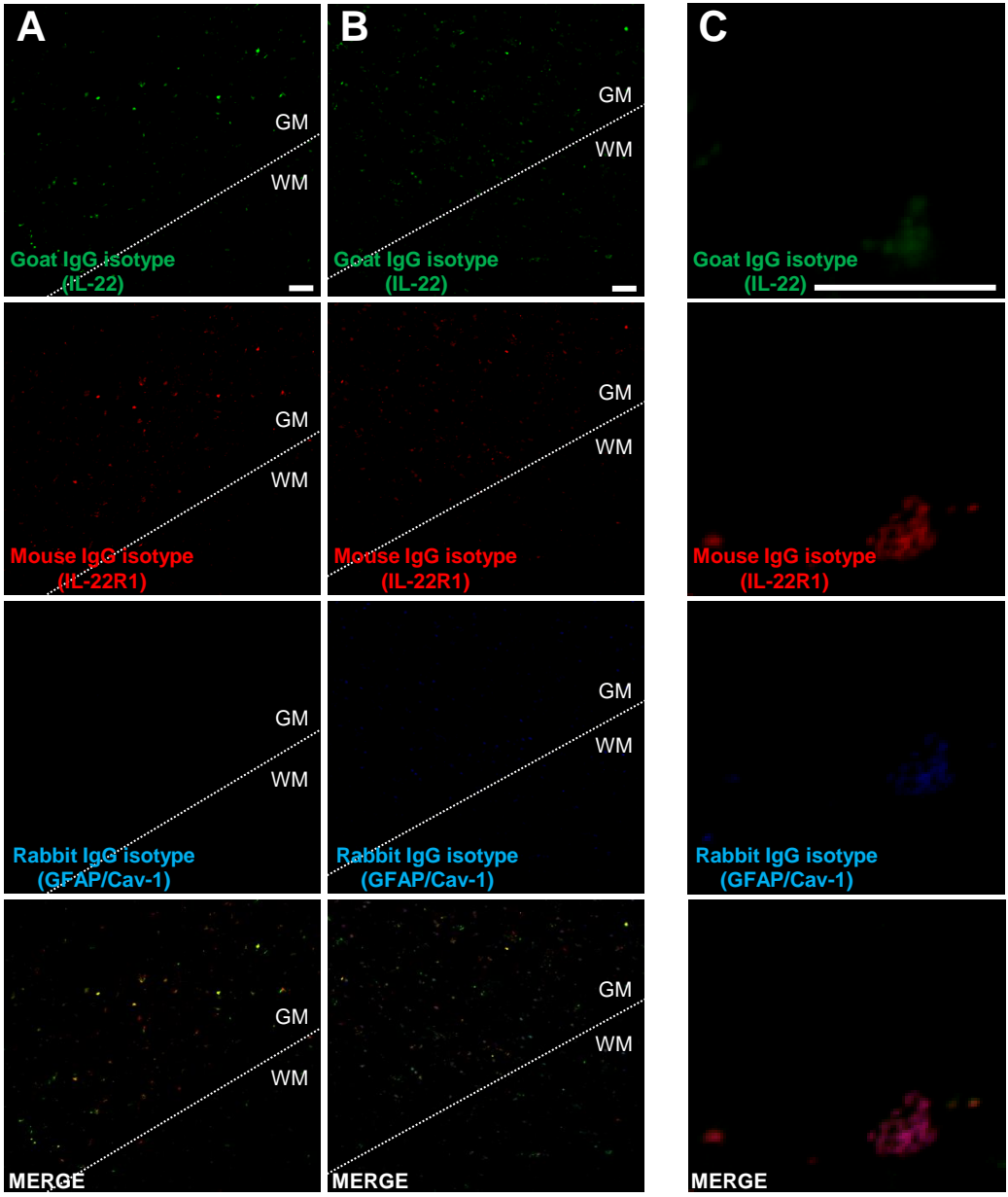

Supplement: Additional file 3: Figure S3. — Peroxidase stainings are specific for IL-22, IL-22R1, GFAP, and Caveolin-1 in autoptic brain tissue from control and MS subjects. Immunohistochemistry peroxidase stainings of isotype controls of goat anti-IL-22, mouse anti-IL-22R1, rabbit anti-GFAP, and rabbit anti-Cav-1 antibodies. Pictures of A, B, C, and D have been acquired at the exact same location as for specific antibodies and mirror exactly panels A, B, C, and D, respectively of Fig. 2. Protocol and images were processed exactly the same way for specific antibodies as for isotype controls. Background is negative for goat and mouse isotype controls (respectively IL-22 and IL-22R1 in Fig. 2). Background for the rabbit isotype control was somehow higher, especially in blood vessels, but remained unspecific and thus did not compromise GFAP and Cavolin-1 specificity. A: study patient B-C2, B and D: study patient B-MS3, C: study patient B-MS5 (Table 2). Scale bar, 50 μm (A–B, ×20; C–D, ×40). GM: gray matter, NAWM: normal appearing white matter, WM: white matter. Representative pictures obtained from the observations of seven control and five MS autopsy samples. [file 12974_2015_335_MOESM3_ESM.pdf]

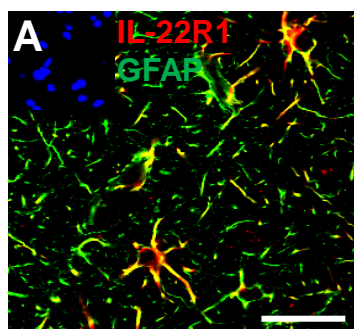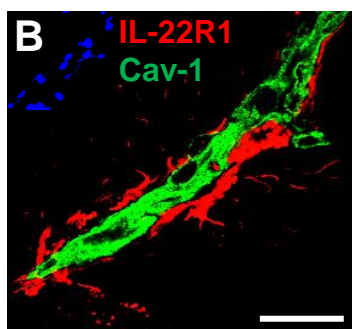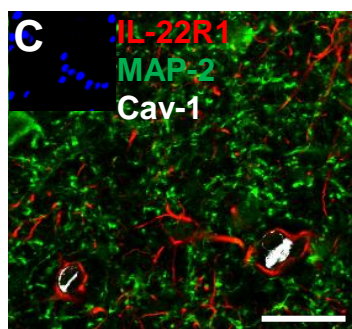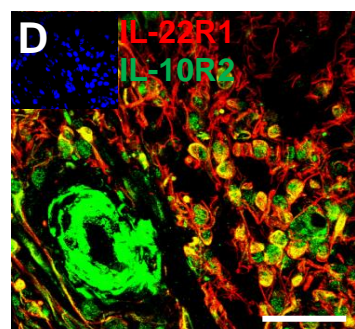

Supplement: Additional file 4: Figure S4. — Immunofluorescence stainings are specific for IL-22, IL-22R1, GFAP and Caveolin-1 in autoptic brain tissue from control patients. Immunofluorescence staining of isotype controls of goat anti-IL-22, mouse anti-IL-22R1, rabbit anti-GFAP and rabbit anti-Cav-1 antibodies. Panel A for A, B for B, and C for C and D, respectively, are duplicates of images of Fig. 3 representing the same tissue location in the brain. Few unspecific background/autofluorescence could be observed, likely due to tissue quality (brain autopsies). The settings of isotype control experiments were exactly the same as for specific antibodies experiments (acquisition parameters and post-processing analyses). Images from panel A and B were taken from B–C6 and C from B–C1 control patient autopsy brain tissues, as for Fig. 3 (Table 2). Bars, 50 μm. GM: gray matter, WM: white matter. Representative pictures obtained from the observations of seven control MS autopsy samples. [file 12974_2015_335_MOESM4_ESM.pdf]

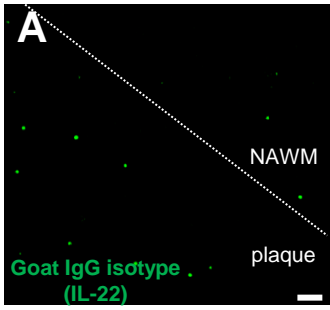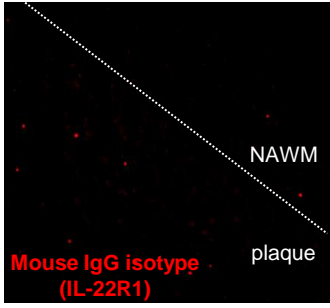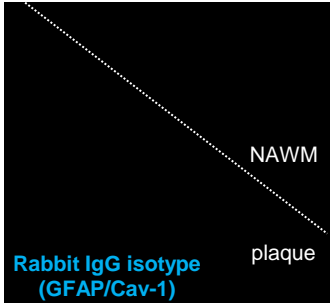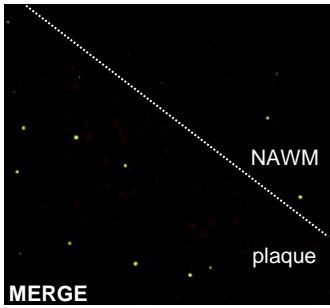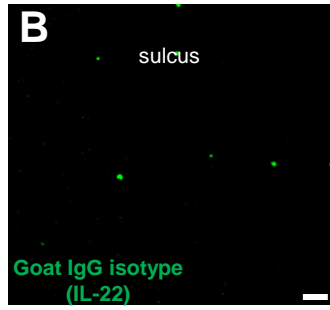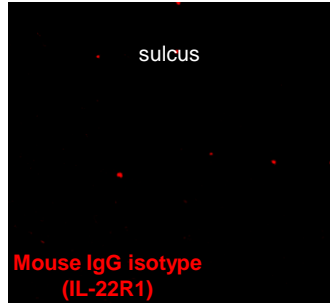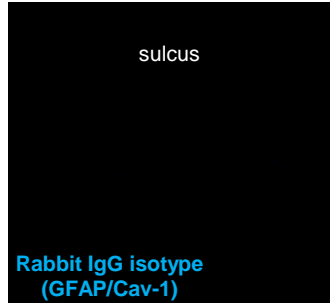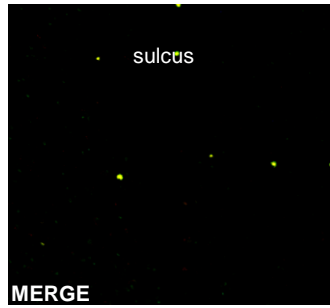

Supplement: Additional file 5: Figure S5. — Immunofluorescence stainings are specific for IL-22, IL-22R1, GFAP and Caveolin-1 in autoptic brain tissue from MS patients. Immunofluorescence staining of isotype controls of goat anti-IL-22, mouse anti-IL-22R1, rabbit anti-GFAP, and rabbit anti-Cav-1 antibodies. Panel A for A and B, and B for C and D, respectively, mirror exactly images in Fig. 4, being from the exact same location and processed identically (same acquisition parameters and post-processing analyses). Thin unspecific autofluorescent area in 488 and 564 nm could be observed. Pictures were taken in patient B-MS3 brain section, again, as for Fig. 4 (Table 2). Bars, 50 μm. GM: gray matter, NAWM: normal appearing white matter, WM: white matter. Representative pictures obtained from the observations of five MS autopsy samples. [file 12974_2015_335_MOESM5_ESM.pdf]

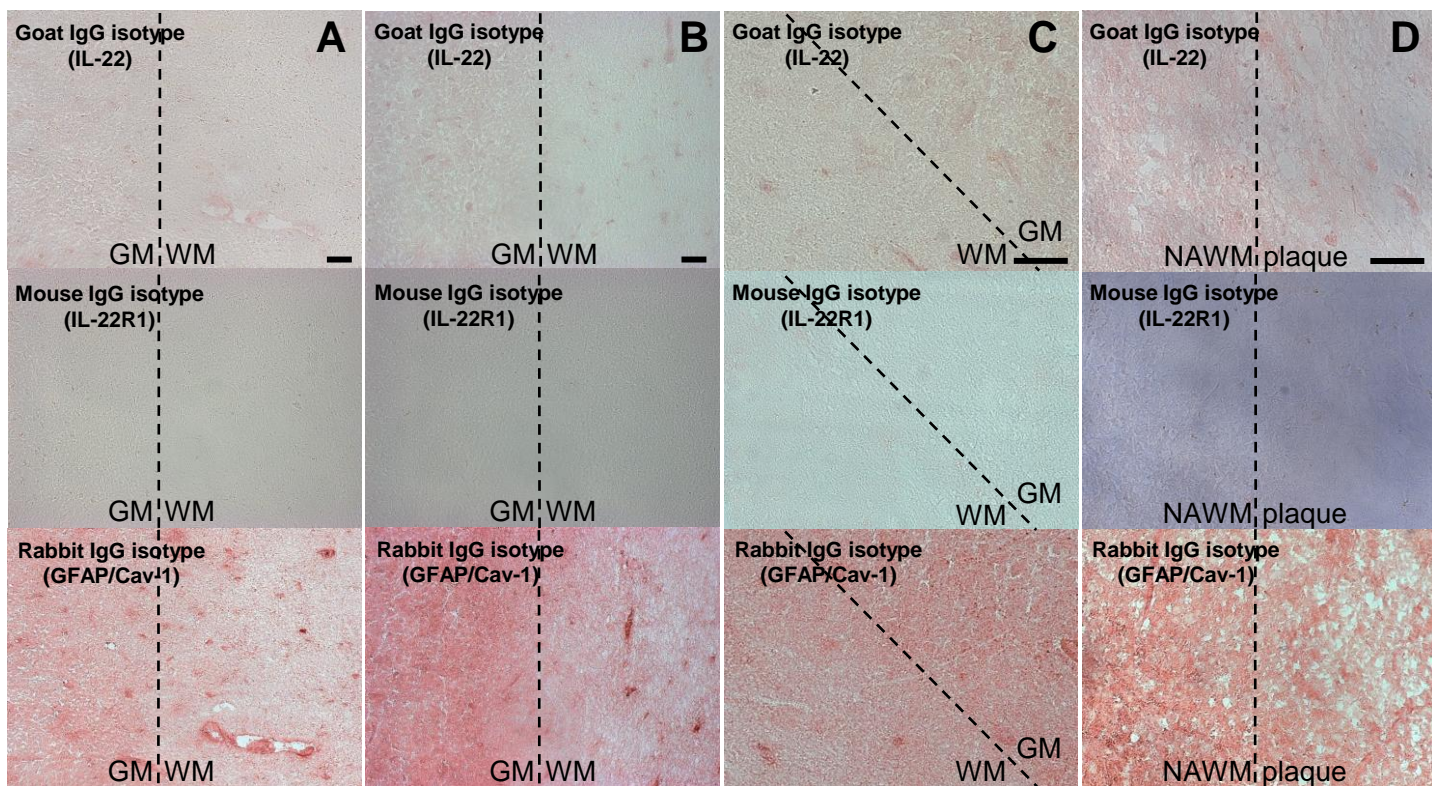

Supplement: Additional file 6: Figure S6. — Colocalization of GFAP and IL-10R2, but not Cav-1 and MAP-2 with IL-22R1 in the brain. Laser scanning confocal microscopy of brain biopsies labeled for IL-22R1 (red) in association with: A) the astrocytic marker, GFAP (green), B) the caveolin-1 endothelial marker (green), C) the caveolin-1 endothelial marker (white) and the neuronal marker, MAP-2 (green), and D) the IL-10R1 subunit receptor marker (green). DAPI staining (blue) is represented in inserts on the upper left side. Images were taken on biopsied brain tissue from control patient L-C5 (A to C) and L-C7 (D) (Table 2). Bar, 50 μm. Representative pictures obtained from the observations of 11 control biopsy samples. [file 12974_2015_335_MOESM6_ESM.pdf]

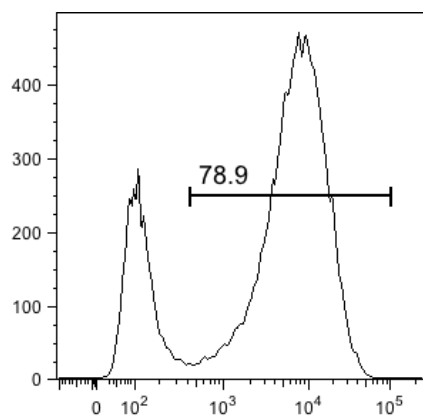

Supplement: Additional file 7: Figure S7. — There is a high degree of colocalization between von Willebrand factor and Caveolin-1, but not between IL-22R1 and any of these two endothelial cell markers in the human brain. Immunofluorescence confocal microscopy images of VWF (blue), IL-22R1 (red), and Cav-1 (green) of two slides, taken at different parts of the brain, of L-C5 study subject (Table 2) are depicted (A and B). Counterstaining was performed with DAPI. Bars, 50 μm. [file 12974_2015_335_MOESM7_ESM.pdf]

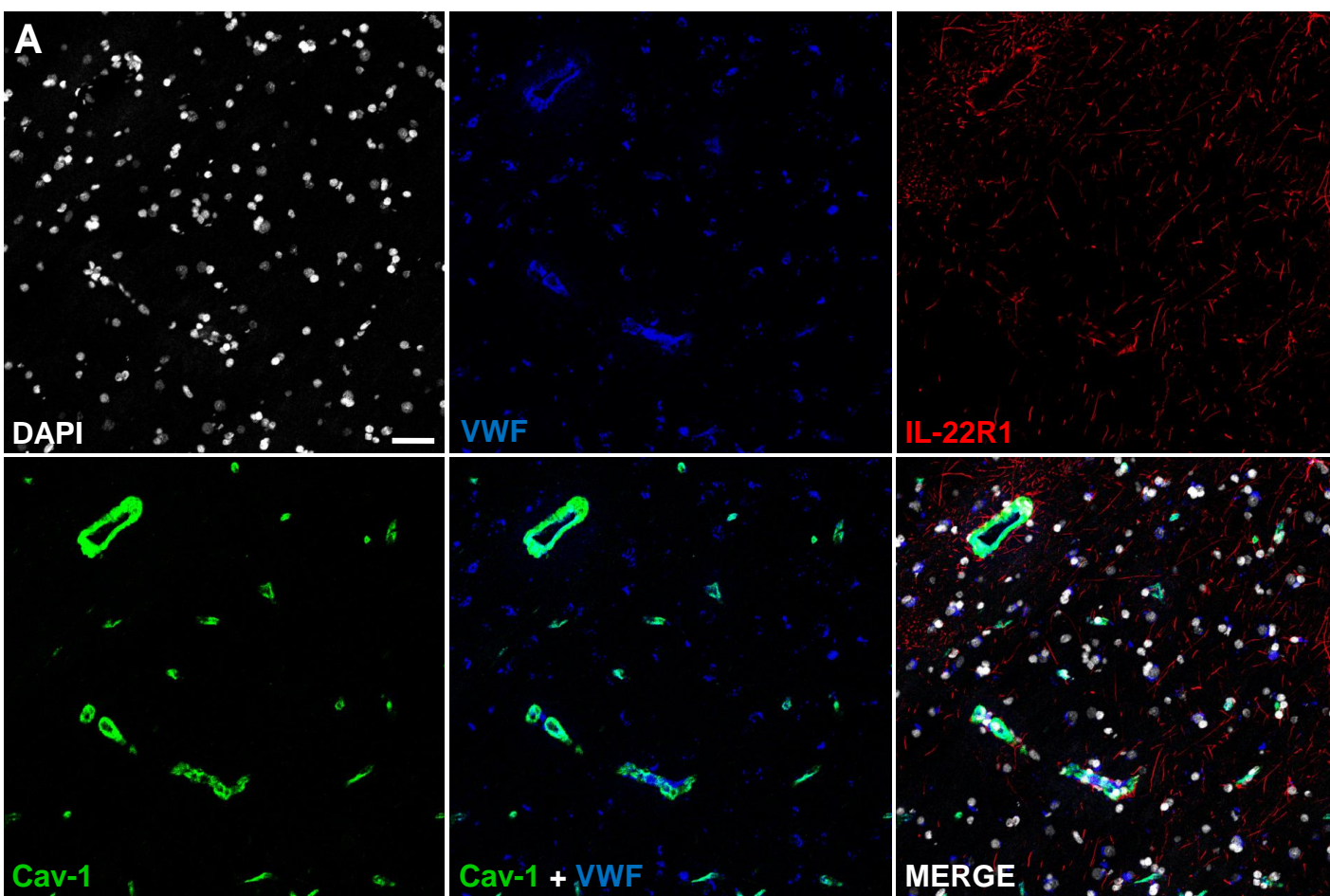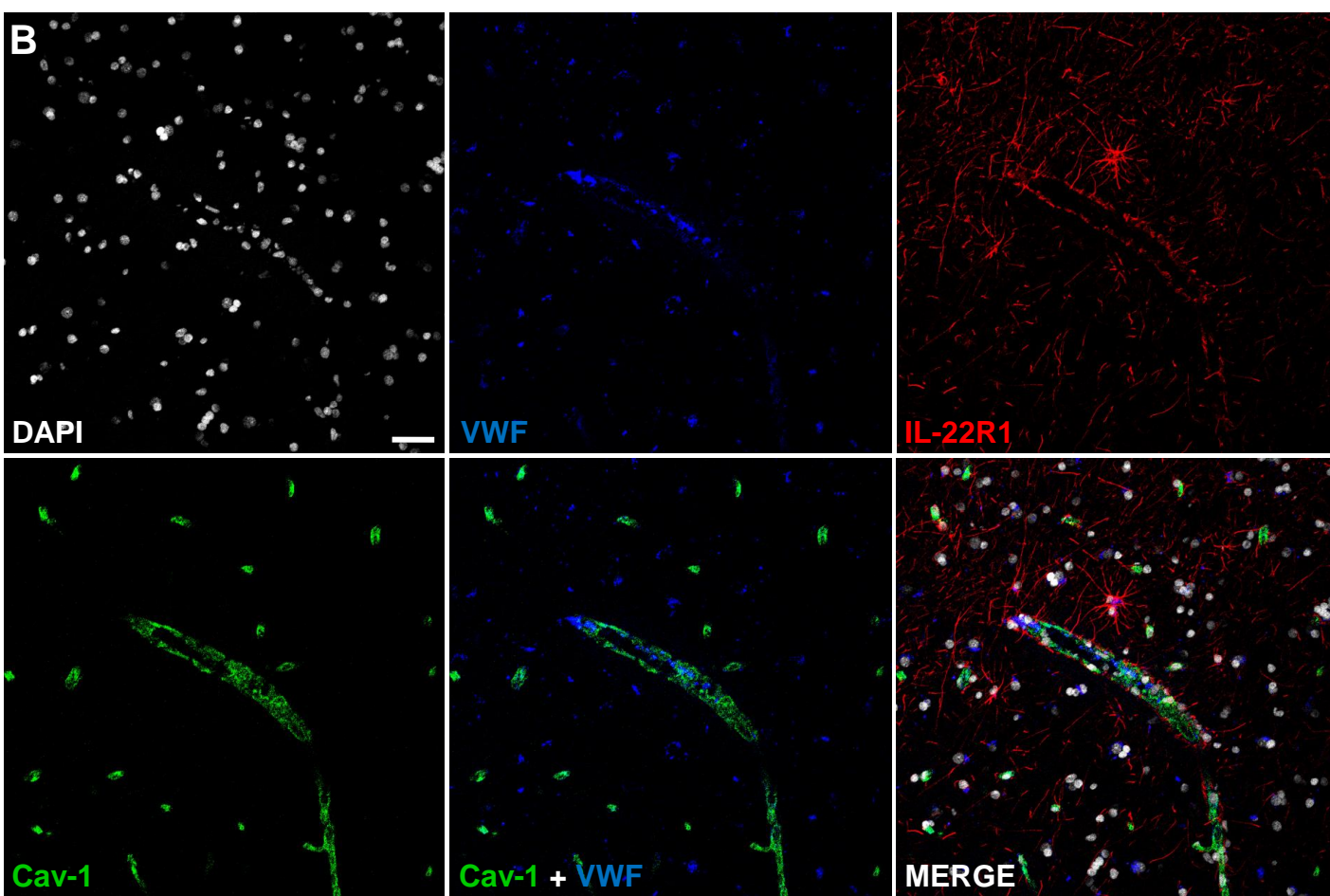

Supplement: Additional file 8: Figure S8. — Large majority of GFAP-positive cells in HA primary astrocytes. Flow cytometry histogram depicting GFAP stained HA primary astrocytes (the bar indicates GFAP-positive cells, in percent). [file 12974_2015_335_MOESM8_ESM.pdf]
